# Features of ectopic lymphoid-like structures in human uveitis

**DOI:** 10.1016/j.exer.2019.107901

**Published:** 2020-02

**Authors:** Simon J. Epps, Natalie Coplin, Philip J. Luthert, Andrew D. Dick, Sarah E. Coupland, Lindsay B. Nicholson

**Affiliations:** a School of Clinical Sciences, University of Bristol, UK; b Institute of Translational Medicine, University of Liverpool, UK; c UCL-Institute of Ophthalmology, UCL, UK; d School of Cellular and Molecular Medicine, University of Bristol, UK; e Liverpool Clinical Laboratories, Royal Liverpool University Hospital, Liverpool, UK

## Abstract

Persistent non-infectious uveitis has a significant morbidity, but the extent to which this is accompanied by inflammation driven remodelling of the tissue is unclear. To address this question, we studied a series of samples selected from two ocular tissue repositories and identified 15 samples with focal infiltration. Eleven of fifteen contained lymphocytes, both B cells (CD20 positive) and T cells (CD3 positive). In 20% of the samples there was evidence of ectopic lymphoid like structures with focal aggregations of B cells and T cells, segregated into anatomically different adjacent zones. To investigate inflammation in the tissue, an analysis of 520 immune relevant transcripts was carried out and 24 genes were differentially upregulated, compared with control tissue. Two of these (CD14 and fibronectin) were increased in ocular inflammation compared to control immune tissue (tonsil). We demonstrate that in a significant minority of patients, chronic persistent uveitis leads to dysregulation of ocular immune surveillance, characterized by the development of areas of local ectopic lymphoid like structures, which may be a target for therapeutic intervention directed at antibody producing cells.

## Highlights

• Active inflammation continues in cases of persistent uveitis.
• Some patients develop ectopic lymphoid-like structure.
• In these cases targeting B cells may be beneficial.

## 1 Introduction

Chronic persistent intraocular inflammation often damages ocular structures producing visual impairment. When the uveitis is undifferentiated, it has been proposed that an ‘uncontrolled, overexuberant immune response’ may contribute to tissue damage (Forrester et al., 2018). Such exuberance could result from heightened immunosurveillance within the tissue. In animal models of inflammatory disease, tissue remodelling and altered immunosurveillance have been demonstrated in many circumstances (Caspi, 2010; Jones et al., 2016). In this paper, we extend a study of human tissue (Epps et al., 2018) to determine whether, when persistent changes are present, they are accompanied by differential expression of inflammatory gene signatures. The changes we identify are relevant in human uveitis, and provide underpinning support for therapies that target B cells.

The healthy retina, like the brain, is immune-privileged and subject to limited immunosurveillance (Shechter et al., 2013), but when uveitis develops, large numbers of leukocytes are found in the eye. Many human autoimmune diseases are associated with chronic persistent accumulation of T and B lymphocytes (Jones et al., 2016; Rao et al., 2017), observations that have in recent years have been extended to include immune privileged CNS tissue (Choi et al., 2012; Pikor et al., 2017). In the most extreme manifestation of this process, the immune cellular infiltrate becomes organized as ectopic lymphoid-like structures (ELS) (Jones et al., 2016). It is plausible that persistent intraocular inflammation might be accompanied by organisation of the immune cell infiltrate, playing a similar role to that seen in chronic inflammatory infiltrate in other organ-specific autoimmune disorders. Persistence and organisation of the immune cell infiltrate into ELS is important, because it influences the prognosis of human disease (Choi et al., 2012; Germain et al., 2015). Understanding whether ELS develop in human uveitis and, if so, what the mechanisms of formation are, may open new avenues for clinical assessment and therapy.

ELS (also known as ectopic lymphoid follicles and tertiary lymphoid organs) are focal aggregations of lymphocytes that develop in non-lymphoid tissues during a chronic disease processes such as autoimmunity, neoplasia and transplant rejection (Aloisi and Pujol-Borrell, 2006; Drayton et al., 2006; Howell et al., 2011; Pitzalis et al., 2014). ELS have features of secondary lymphoid organs, including focal aggregations of B cells and T cells (in adjacent but anatomically distinct areas of tissue) and follicular dendritic cell networks resembling the germinal centre-containing lymphoid follicles of secondary lymphoid organs (SLOs). These structures have many important properties that have been described in both experimental and human disease. They are sites of autoantibody production (Corsiero et al., 2016), whose presence has been correlated with response to biologic therapy in rheumatoid arthritis (Canete et al., 2009). They have also been correlated with clinical prognosis and neurodegeneration in multiple sclerosis (Choi et al., 2012; Magliozzi et al., 2007), and they have an influence on anti-tumour immunity (Germain et al., 2015).

In uveitis B cells and plasma cells have been identified within the human aqueous and vitreous humor and diffuse and focal infiltrates observed in the uveal tract and retina by immunocytochemistry (George et al., 1997; Kim et al., 1987; Lubin et al., 1980; Sabates et al., 1979). ELS have been described in the retina of R161H mice with experimental uveitis (Kielczewski et al., 2016) and in the iris, ciliary body and retina of horses with equine recurrent uveitis (Deeg et al., 2002; Kleinwort et al., 2016), but they have not been specifically reported in humans. We therefore characterized the immune cell infiltrate in a series of human uveitis specimens. We show that features of ELS are present in a minority of these specimens and these features are accompanied by the upregulation of immune associated genes including those related to B cell function.

## 2 Materials and methods

Eye pathology databases from the Liverpool Ocular Oncology Biobank (Liverpool, UK; R + D Number: 4116) and Moorfields Biobank (London, UK. Ref: 10/H0106/57-2015ETR47), were searched with the ethical approval of the local institutional review boards, using the keyword uveitis. From 200 formal NHS histopathology reports, made over a period of 10 years, we identified 15 samples of enucleated or eviscerated tissue described as having visible lymphocytes within the uvea or retina on haematoxylin- and eosin-stained (H&E) sections. These formalin-fixed paraffin-embedded (FFPE) enucleated or eviscerated human eyes were obtained. The limited nature of the available clinical information meant that a prior history of infections could not be completely excluded. Two 4 μm sections were examined by immunohistochemistry (IHC) for CD3^+^ T cells and CD20^+^ B cells. If CD20^+^ cells were present in the uvea or retina, additional IHC was performed to identify whether other features of ELS were present using four further serial sections stained for the lymphoid follicle markers CD21 (follicular dendritic cells, FDCs), CD23 (follicular B cells, FoB), BCL6 (B-cell lymphoma 6 protein) and AID (activation-induced cytidine deaminase). Sections were de-waxed and subjected to heat-induced epitope retrieval (with low or high pH antigen retrieval solution according to manufacturer's technical data sheet for each antibody) using EnVision FLEX + Target Retrieval solution and PT Link (Dako; Glostrup, Denmark). Samples were incubated with peroxidase blocking solution (Dako) for 5 min to block endogenous peroxidase activity followed by a 30-min incubation with primary antibody (see Table S1). All primary antibodies were obtained from Dako with the exception of anti-AID which was obtained from Life Technologies (now ThermoFisher Scientific, Waltham, USA). Signal from primary antibody was amplified by incubating samples with a species-appropriate linker molecule for 15 min where indicated in Table S1. Primary antibody binding was visualised using the appropriate secondary antibody conjugated with HRP (Dako) and diaminobenzidine chromogen (Dako). All sections were counterstained using haematoxylin and sealed under a coverslip with Depex Polystyrene prior to viewing. IgG isotype control antibodies or primary antibodies omitted were used for negative controls. Control tissue without evidence of intraocular inflammation was examined in parallel.

## 3 Demographic and clinical data

Demographic and clinical data for each patient were obtained from the limited clinical information supplied to the histopathologist with each sample and summarised according to IUSG (Deschenes et al., 2008) (with respect to aetiology of uveitis) and SUN classifications (Trusko et al., 2013) (with respect to anatomical classification of uveitis) (Table 1). The demographic data obtained were age at time of tissue collection and sex. Clinical data specified disease aetiology, course and duration. The anatomical classification of disease into anterior, intermediate, posterior or panuveitis categories was made on the basis of histopathological appearances as well as the limited clinical information available from the pathologist's report. No other clinical documents were available.

**Table 1:** Patient characteristics.

Patient	Age	Sex	Sample type	Clinical classification of uveitis (IUSG, 2005)	Anatomic classification of uveitis	Notes
**P1**	28	F	Enucleation	Non-infectious, no known systemic association	Panuveitis	Idiopathic uveitis. Enucleation for blind painful eye.
**P2**	29	F	Enucleation	Non-infectious, no known systemic association	Panuveitis	History of scleritis and episcleritis in addition to uveitis. Enucleation for blind painful eye.
**P3**	26	F	Enucleation	Non-infectious, no known systemic association	Panuveitis	Idiopathic uveitis complicated by glaucoma
**P4**	17	M	Enucleation	Non-infectious, known systemic association	Panuveitis	Clinical diagnosis of probable Behcet's disease-associated uveitis, no systemic features of Behcet's disease, HLA B51+. Enucleation for blind painful eye.
**P5**	61	F	Enucleation	Non-infectious, no known systemic association	Panuveitis	Idiopathic uveitis. Enucleation for suspected sympathetic ophthalmia following multiple intraocular surgeries but histological features not diagnostic of SO.
**P6**	N/A	F	Evisceration	N/A	Panuveitis	N/A
**P7**	N/A	F	Enucleation	Non-infectious, no known systemic association	Intermediate	Idiopathic uveitis
**P8**	38	M	Enucleation	Non-infectious, known systemic association	Panuveitis	Behcet's disease-associated uveitis
**P9**	74	F	Enucleation	Non-infectious, no known systemic association	Panuveitis	Idiopathic uveitis
**P10**	70	F	Enucleation	Non-infectious, known systemic association	Panuveitis	Systemic granulomatosis with polyangiitis (Wegener's granulomatosis) with scleritis and uveitis
**P11**	26	F	Enucleation	Non-infectious, known systemic association	Anterior	Juvenile idiopathic arthritis-associated uveitis
**P12**	34	M	Evisceration	Non-infectious, no known systemic association	Posterior	Idiopathic uveitis
**P13**	33	M	Evisceration	Non-infectious, no known systemic association	Posterior	Idiopathic uveitis
**P14**	77	F	Enucleation	Non-infectious, no known systemic association	Panuveitis	Idiopathic uveitis
**P15**	50	M	Enucleation	Non-infectious, no known systemic association	Panuveitis	Idiopathic uveitis

Formalin-fixed paraffin-embedded enucleated or eviscerated human eyes, from 15 patients with uveitis, were obtained from the Liverpool Ocular Oncology Biobank (Liverpool, UK) and Moorfields Biobank (London, UK) with ethical approval. Samples were identified by reviewing histopathology reports from each institution and selected if reported to contain features of chronic inflammation i.e. visible lymphocytes within the uvea or retina on haematoxylin- and eosin-staining. All patients had a persistent, chronic clinical course of disease. The duration of clinical disease could not be ascertained from the limited notes available and neither could we completely rule out a history of ocular infections.

## 4 Immunohistochemical characterisation of B cell infiltrate

**Fig. 1:**
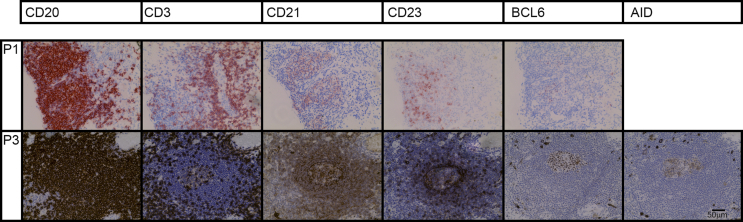
Ectopic lymphoid-like structures develop in patients with persistent uveitis. Serial sections from two individual eyes are shown (P1; upper panel and P3; lower panel). Focal aggregates of CD20 ^+^ cells encompass areas of CD21, CD23 and BCL6 immunostaining. CD3^+^ cells are present adjacent to the focal aggregates of CD20 ^+^ cells with, in addition a few CD3^+^ cells scattered within the area of CD20 ^+^ immunostaining. There is an area of AID expression in a section from patient 3. The location of the lesion from P1 is identified with an arrow in Supplementary Fig. 1A. Where staining is negative, no image is shown.

Following immunostaining, the tissue sections were examined by light microscopy to classify the predominant pattern of CD20^+^ B cell infiltration and record the tissue(s) affected (Table 2). If CD20^+^ cells were present in either a diffuse or focal infiltrate, further IHC was performed for CD21, CD23, BCL6 and AID. Samples were then categorised as having: (i) sparse infiltrate; (ii) diffuse infiltrate; (iii) focal infiltration; (iv) focal infiltration with CD20/CD3 segregation and CD23^+^ cells, CD21 ^+^ network(s), and BCL6^+^ cells within areas of CD20^+^ focal aggregation; (v) as (iv) with added AID positive cells (see Fig. 1).

**Table 2:** Characteristics of CD20 infiltrate.

Subject	CD20 ^+^ infiltrate				Tissue(s) affected	Notes
	Diffuse	Focal	ELS like	AID positive		
**P1**	+	+	+	+	Iris, ciliary body, choroid	B cell follicles in iris, ciliary body, and choroid; x/y follicles displaying GC markers
**P2**	+	+	+	–	Choroid, sclera	B cell follicles in choroid and sclera; x/y follicles displaying GC markers
**P3**	+	+	+	+	Choroid	B cell follicles in choroid; x/y follicles displaying GC markers
**P4**	+	+	–	–	Iris, ciliary body, choroid	Significant CD20 ^+^ infiltrate in iris, ciliary body and choroid with focal aggregations of CD20 ^+^ cells but no other features of ELS
**P5**	+	–	–	–	Iris, ciliary body and choroid	Dense diffuse infiltrate in iris, ciliary body and choroid but no focal aggregations of CD20 ^+^ cells or other features of ELS
**P6**	+	+	–	–	Choroid	CD20 ^+^ infiltrate present in choroid with focal aggregations of CD20 ^+^ cells proximal to focal aggregations of CD3^+^ cells but no clear segregation into B and T cell zones
**P7**	+	–	–	–	Iris, choroid	Sparse diffuse CD20 ^+^ infiltrate in choroid
**P8**	+	–	–	–	Choroid	Sparse diffuse CD20 ^+^ infiltrate in choroid
**P9**	+	+	–	–	Choroid	Small focal CD20 ^+^ aggregations in choroid
**P10**	–	–	–	–	N/A	No CD20 ^+^ infiltrate present
**P11**	–	–	–	–	N/A	No CD20 ^+^ infiltrate present
**P12**	–	–	–	–	N/A	No CD20 ^+^ infiltrate present
**P13**	+	+	–	–	Retina, choroid	2 small focal aggregations of CD20 ^+^ cells, 1 in retina (appears perivascular) and 1 in choroid
**P14**	+	+	–	–	Choroid	3 small focal aggregations of CD20 ^+^ cells in choroid
**P15**	–	–	–	–	N/A	

Infiltrate was classified on the basis of CD20 infiltration as diffuse or focal. ELS was defined as: Focal CD20 ^+^ aggregations with CD20/CD3 segregation, CD21 ^+^ network(s), CD23 ^+^ cells within areas of CD20 ^+^ focal aggregation and BCL6+ cells within area of CD20 ^+^ aggregation. Within ELS like tissue, focal aggregates were tested for AID expression.

## 5 Image analysis

Tissue sections were analysed on a Leica DM1000 light microscope (Leica Microsystems, Wetzlar, Germany) using Brightfield imaging. Images were captured using an EVOS XL Cell Imaging System (ThermoFisher Scientific, Waltham, USA) and prepared in Photoshop CS6 for publication.

## 6 Gene expression and statistical analysis

Three samples of RNA were prepared from each specimen and the quality of RNA was tested using a Nanodrop and Agilent Bioanalyser. We studied the ocular tissue of patients with ELS (labelled ELS), ocular tissue with cell infiltrate (labelled INF), ocular tissue negative for cell infiltrate (designated NEG) from subjects with persistent uveitis and normal ocular tissue (labelled NOT) obtained post-mortem for donors with no history of ophthalmic disease. A single sample was prepared from tonsil (TON).

RNA expression was assessed by Nanostring using the nCounter® Human Immunology v2 Panel. This profiles 594 genes; 579 immunology-related human genes and 15 internal reference controls. Pre-processing was carried out to exclude genes with low or undetectable levels of expression from statistical analysis which reduced the set of genes that we compared to 520.

Analysis was carried out in the R environment (v3.4.3) using a linear model for gene expression. Unsupervised clustering of the samples demonstrated that one sample, from a subject with infiltration negative uveitis, had a gene expression pattern that resembled that of samples from eyes with ELS and infiltrate. The subject had a diagnosis of uveitis associated with juvenile idiopathic arthritis (JIA), and this sample was removed from further analysis because the clinical diagnosis and molecular diagnosis were inconsistent. Normalisation of nanostring data was carried out in the limma package, using voom implementing cyclic loess (Law et al., 2014). Differential gene expression was assessed using the limma package (decideTests: significance level: corrected P < 0.05, false discovery rate <0.05) (Ritchie et al., 2015), to compare samples of ocular tissue from patients with infiltration and ELS with samples from normal ocular tissue plus samples from patients without infiltrative disease. (Comparison between samples from patients with ELS and patients with only infiltration did not identify any significant differences (four samples compared with two samples). This likely reflects a lack of statistical power with this low number of replicates. Therefore, absolute expression levels were compared as the geometric mean of nanostring counts in the different sample groups.

## 7 Results

We examined FFPE specimens from 15 patients with a history of uveitis that had undergone either evisceration (n = 3) or enucleation (n = 12) and whose histopathology report included a finding of visible lymphocytes. The patient age range was 17–77 years (mean 43 years, median 34 years) and the male to female ratio was 1:3 (Table 1). All patients had a history of chronic non-infectious uveitis, with the majority of patients having panuveitis (n = 11) and smaller numbers having anterior (n = 1), intermediate (n = 1) or posterior (n = 2) uveitis. Non-infectious uveitis with no known systemic association (undifferentiated) constituted the greatest part of the case series (n = 11), with the remainder of the cases being associated with Behçet's disease (n = 2), JIA (n = 1) or systemic granulomatosis with polyangiitis (n = 1). Although the patient with JIA had no CD20 infiltrate, unsupervised clustering of gene expression indicated an inflammatory gene signature, and this case was excluded from the analysis of differential gene expression.

**Supplementary Fig. 1:**
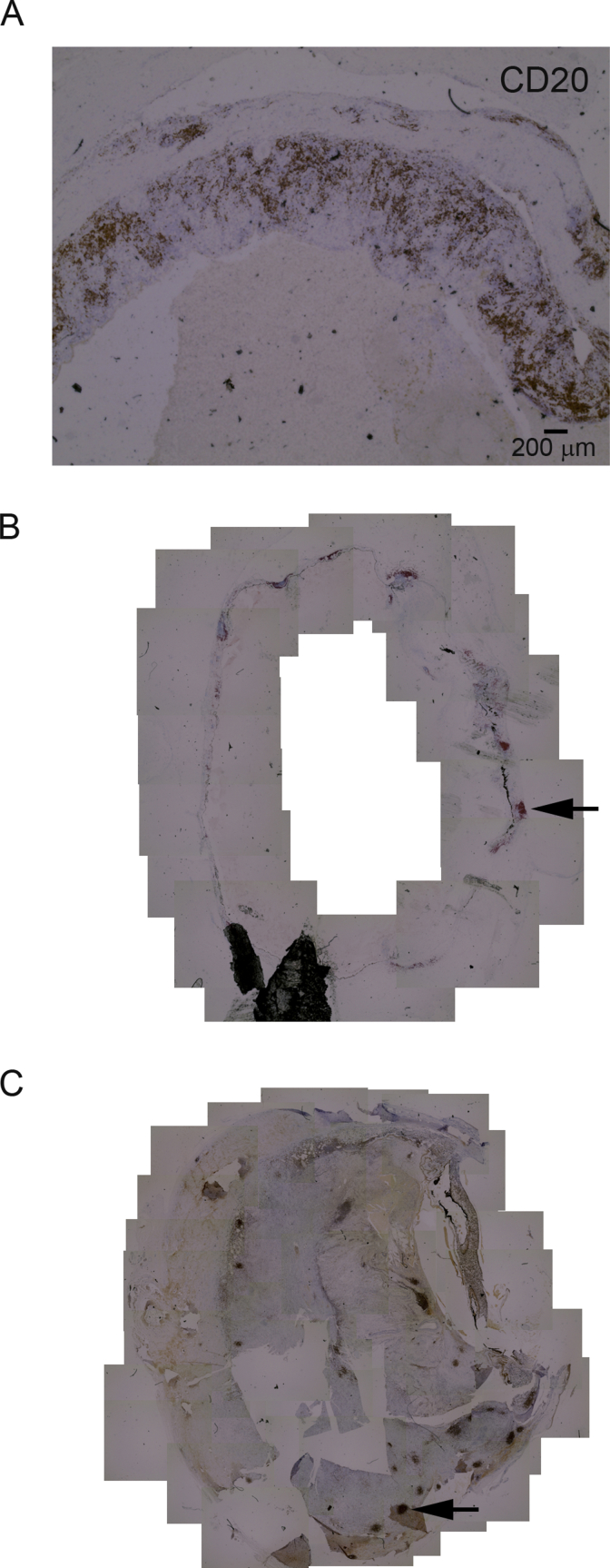
CD20 staining showing diffuse infiltration of CD20 ^+^ cells within the uvea of patient 4 (A), and composite low magnification (×40) images of P1 (B) and P2 (C) showing focal aggregations of CD20 ^+^ cells within the uvea in patients 1–2 (B,C) and. Arrows in supplementary figures indicate examples of focal aggregations of CD20 ^+^ cells highlighted in the main text (Fig. 1, Fig. 2)

CD3^+^ T cells and CD20^+^ B cells were detected together in 11 out of 15 samples (73%): of the 11 samples containing B cells 2 had a sparse CD20^+^ infiltration, 1 had a dense diffuse CD20^+^ infiltrate (patient 5, supplementary Fig. 1A), and 8 had focal aggregates of CD20^+^ cells (e.g. patients 1 and 2, Supplementary Figs. 1C and 1C respectively). Of the 8 samples with focal CD20^+^ aggregates, 5 also had areas of diffuse CD20^+^ infiltrates (e.g. patient 2 represented in Supplementary Fig. 1C).

In all 11 samples where B cells were present, these cells were seen in the choroid; Fig. 1 shows a representative dense focal CD20^+^ infiltrate typical of those found in patients 1 and 3. In 4 of the 11 samples containing B cells, the anterior uvea (iris, ciliary body or both) was also involved. Only in 1 of the 11 samples with B cell infiltrate were these cells seen within the retina (patient 13); patient 2 also had a focal aggregation of B cells within the sclera. Table 2 summarises the tissue distribution of the B cell infiltrate for each patient.

**Fig. 2:**
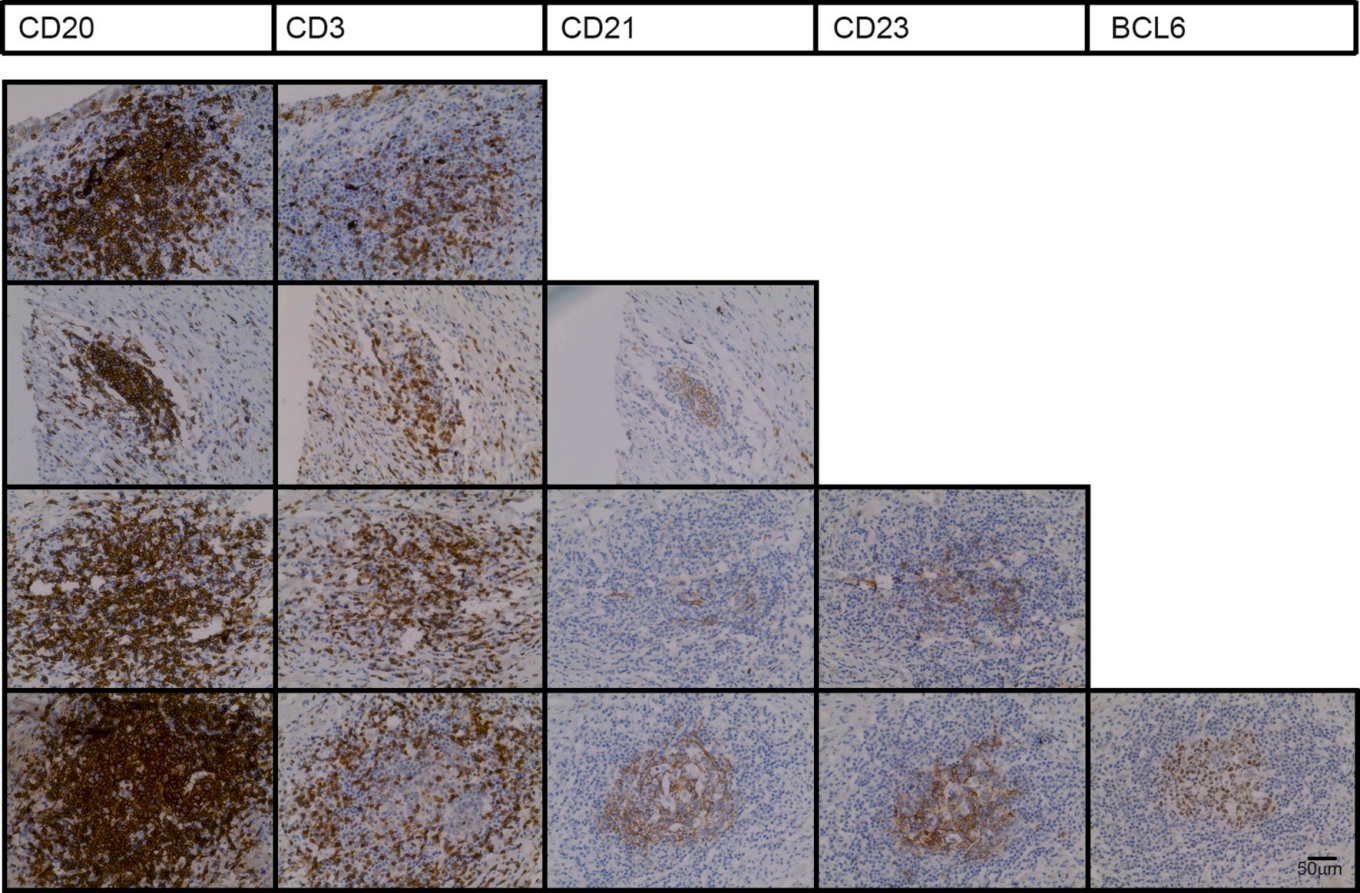
ELS development in a single eye is asynchronous. Serial sections from patient P2 were examined for features of ELS. Positive staining in a representative selection of lesions is shown. The full range of B cell markers were only present in a minority of cell aggregates. Where staining is negative, no image is shown. The images within the lowest panel are taken from the lesion marked with an arrow in Supplementary Fig. 1C.

For all samples where a diffuse or focal CD20^+^ infiltrate was evident, further immunohistochemistry was performed on serial sections that were examined for CD21 (identifying follicular dendritic cells), CD23 (identifying follicular B cells), BCL6 and AID (both of which are expressed by germinal centre B cells). These markers were not detected in any patient that had only a diffuse B-cell infiltrate. Where there was focal infiltration, positive staining was detected in a subset of patients and within each eye in only a subset of lesions. Three patients (20% of the total case series of 15 patients or 27% of the 11 patients with a B cell infiltrate) had features of ELS within immune cell infiltrate i.e. focal aggregations of CD20^+^ cells and CD3^+^ cells segregated into anatomically distinct but adjacent zones, with a CD21^+^ follicular dendritic cell (FDC) network, CD23^+^ cells and BCL6 staining in the B cell zone (Figs. 1 and 2). In two of these (patients 1 and 3), AID was also detected (Fig. 1), which provides further evidence of local B cell receptor rearrangement.

**Fig. 3:**
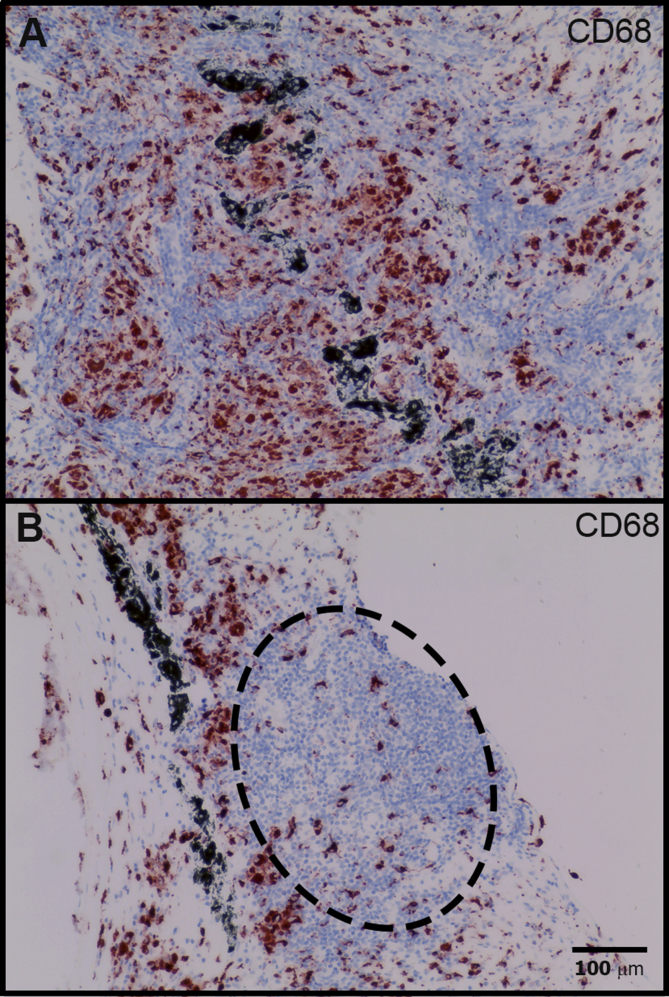
Macrophages are widely distributed in chronic lesions. IHC staining of P1 to detect CD68 demonstrates a diffuse infiltration, dense in places (A). These cells are sparse in regions of lymphoid aggregation (oval area with dashed margin) and do not have features of granulomata (B).

Staining with anti-CD68 to assess the distribution of macrophages revealed large numbers of CD68^+^ cells in the ocular tissues (Fig. 3A), but only small numbers within the lymphoid aggregates resembling ELS (Fig. 3B). This indicates that these lymphoid aggregates were not simply non-caseating granulomata (which are a known feature of ocular inflammation, e.g. in sarcoidosis).

**Supplementary Fig. 2:**
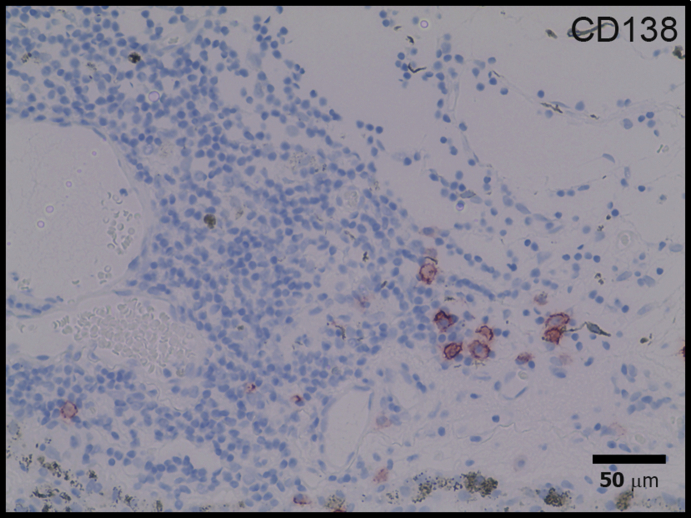
CD138 + plasma cells are visible in the areas surrounding lymphoid aggregates of P1.

It was evident that within a single eye there was a range of different types of immune cell infiltrate (Fig. 2). This extended from simple focal aggregates of lymphocytes, through focal aggregates of lymphocytes with an FDC network, to focal aggregates of lymphocytes containing an FDC network and germinal centre markers (BCL6 and/or AID). CD138^+^ (syndecan-1 positive) plasma cells were also visible in the areas surrounding lymphoid aggregates (Supplementary Fig. 2).

**Fig. 4:**
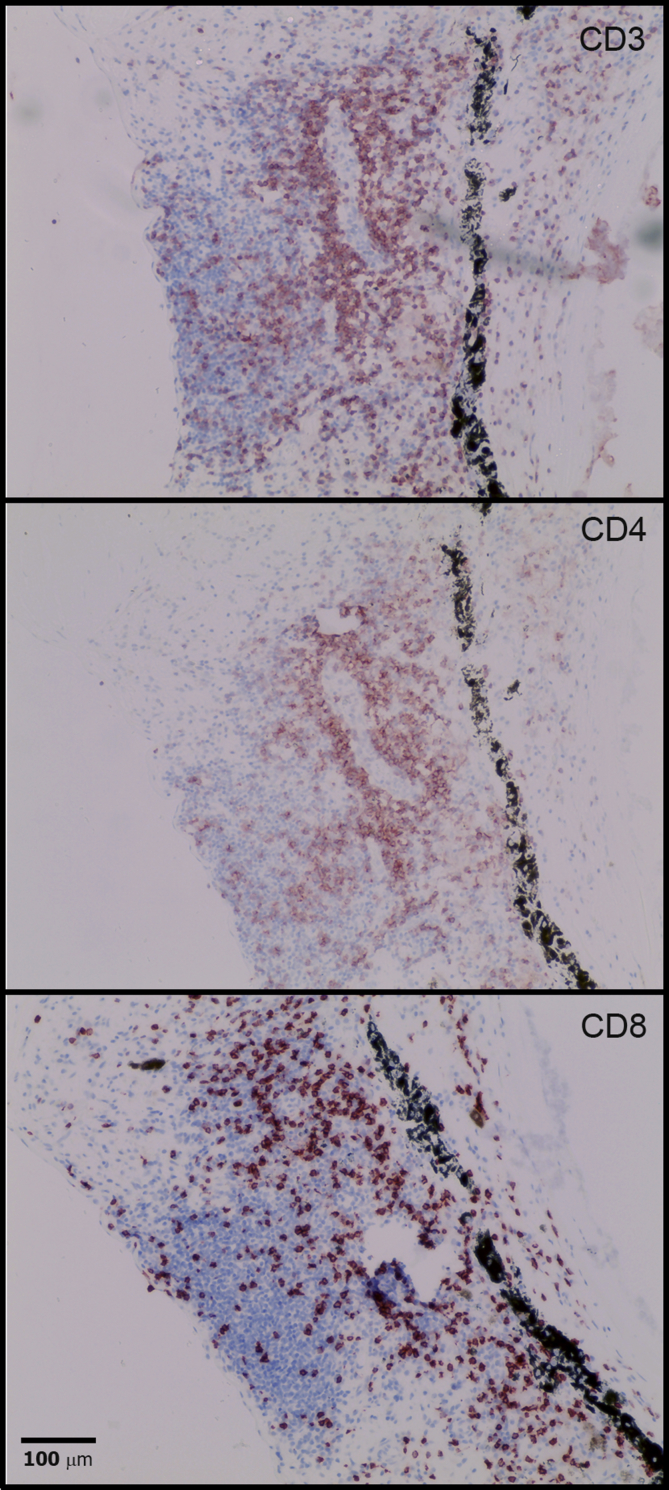
Lymphocytes of different phenotypes are present in chronic lesions. IHC staining of P1, to identify the phenotype of CD3 positive lymphocytes, confirms that both CD4 positive and CD8 positive cells are present in significant numbers.

All the eyes with infiltrating B cells (11/15) also contained CD3^+^ cells. Selected lymphoid aggregates were stained with CD4 and CD8 lymphocyte markers (Fig. 4). Significant numbers of CD4^+^ and CD8^+^ cells were present within the lymphoid aggregates and this was the case whether or not there was evidence of ELS. This is consistent with data from animal models of autoimmune uveitis that find that T cell infiltration with CD4^+^ and CD8^+^ cells is an early feature in the development of disease (Boldison et al., 2014, 2015).

**Fig. 5:**
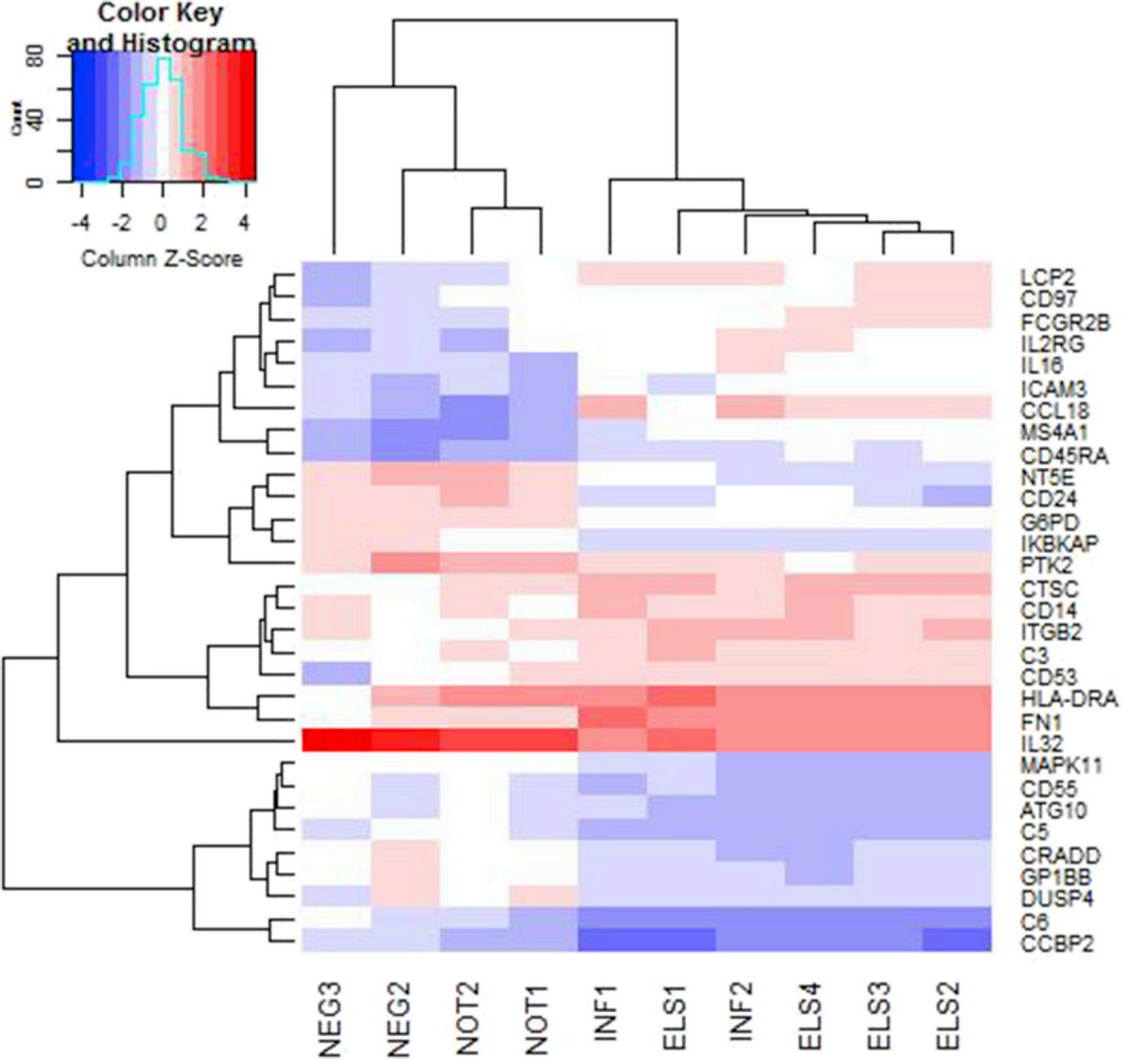
Genes that were differentially expressed between control ocular tissue (NEG and NOT) and tissue with inflammation (ELS and INF) were grouped by unsupervised clustering. There is segregation of control versus inflamed eyes. The heatmap illustrates that these genes fall into a number of related groups. Heatmap was prepared using heatmap.2, part of the gplots package (Warnes et al. 2019).

Differential gene expression of a panel of immune relevant genes was carried out on mRNA prepared from selected tissues, comparing those with (INF and ELS) and without infiltration (NOT and NEG). This identified 32 genes (corrected P < 0.05, false discovery rate <0.05) that varied significantly between the two groups. Comparing any tissue that contained B-cell infiltrate (i.e. INF plus ELS) with normal and non-infiltrated tissue, identified 16 genes that were upregulated and 16 that were downregulated. When samples from tissue with ELS only were compared to tissue without infiltrate, 31 genes were identified (16 upregulated and 15 downregulated). When these genes were assessed in unsupervised clustering of all the samples, control and diseased tissue were clearly segregated, although ELS and inflamed tissues were not (Fig. 5). The genes also clustered into distinct groups with different patterns of expression. We did not identify any genes differentially expressed when we compared inflamed tissue and tissue from patients with ELS. This is likely to be due to (a) insufficient power because of the small sample size and (b) that we were studying whole tissue not just the ELS themselves.

As anticipated from the histology, and expected because we used a curated human immunology gene panel, genes that were significantly upregulated in tissue from patients with infiltrate compared with control samples were predominantly associated with the immune response. Nineteen genes were upregulated compared with control (NEG plus NOT) samples. Thirteen were identified both in ELS alone and ELS plus INF samples, and 6 identified in one or other of the two comparisons. These are summarised in Table 3 and shown as a reference with the level of expression of the same genes measured in a single tonsil sample. Upregulation in this set of genes was on average 20-fold greater in the inflamed tissue compared with the controls. The genes represent a broad cross-section of different immune functions, including genes associated with antibody generation, T cell activation and signalling, innate immunity and chemokine signalling. It was also found that there was significant upregulation of the cell matrix protein fibronectin. For the genes, that were found to be differentially expressed in inflamed ocular tissue, their level of expression in ocular tissue was generally lower than that measured in the tonsil (Table 3). The exceptions were CD14 and fibronectin, which were upregulated in samples from persistently inflamed eyes, but not expressed at high levels in tonsillar tissue.

**Table 3:** Genes significantly upregulated in ocular tissue with persistent inflammation.

Gene	Function	ELS	INFIL	NOT	TON	Summary
ARHGDIB*	T cell growth and signalling	616	736	53	1798	Interacts with VAV1 and Src. Expression may limit metastasis
**CD53**		399	467	14	1331	Contributes to CD2 signalling. Mutation leads to immunodeficiency
**IL2RG**		201	213	7	735	Common gamma chain used by lymphocyte growth factor receptors
**LCP2**		224	263	9	508	Slp-76
CD45R0*		208	248	13	600	Memory T cell marker; found in ERU follicles
**CD45RA**		65	69	2	783	Naive T cell marker
**HLA-DRA**	Antigen processing and presentation	2639	2656	130	4915	MHC II alpha chain
CTSS*		753	900	39	750	Cathepsin S
**CTSC**		612	678	40	679	Cathepsin C
**FN1**	Matrix interactions	2345	2690	51	105	Fibronectin
**ICAM3**		125	135	6	302	CD50 ligand for LFA-1
**ITGB2**		592	612	32	658	CD41; Integrin alpha chain 2b. Fibrinogen receptor in platelets
**CD14**	Innate immunity	567	645	34	140	TLR4 co-factor
C3**		511	453	31	486	Complement factor 3
CD97**		174	181	9	310	GPCR family, interacts with decay activating factor
FCGR2B**	B cell function	278	232	13	93	Inhibitory receptor CD32B, expressed on B cells
**MS4A1**		120	119	2	2281	CD20; target of Rituximab
**CCL18**	Cell localisation	242	457	3	471	Produced by APCs of the innate immune system, attracts non-Foxp3 pos Tregs. CCR8 probably the ligand
**IL16**		173	185	6	863	Chemoattractant for CD4 cells

Genes upregulated in ocular tissue with persistent inflammation (P < 0.05; FDR<0.05), comparing control samples with all inflamed samples or only samples with ELS. Gene expression measured by nanostring counts from ocular tissue with ectopic follicle like structures (ELS), immune cell infiltrate (INFIL), non-infiltrated plus normal tissues (NOT) and tonsil (TON). *Genes only identified vs all inflamed tissue, ** genes identified only vs ELS. All other genes identified in both analyses. Data are the geometric mean of the gene expression measured using the nCounter array except TON which is a single measurement from tissue. Differential gene expression was assessed following normalisation using the voom function in limma, implementing cyclic loess (Law et al., 2014; Ritchie et al., 2015).

The fold-changes of the downregulated genes were small, which may represent an insensitivity to changes from an already low baseline, and these associations were not tabulated further.

## 8 Discussion

Non-infectious uveitis is most often an autoimmune or autoinflammatory disorder (Lee et al., 2014) and has been modelled in many experimental animal systems, most prominently in rodents. Animal models of autoimmune uveitis fall into two distinct groups (Caspi, 2010): firstly, those where uveitis is induced by injection of a retinal protein with an adjuvant resulting in a CD4^+^ T lymphocyte-mediated intraocular inflammation; and secondly, those in which uveitis arises spontaneously such as the R161H model, where mice are transgenic for a T cell receptor specific for the retinal antigen interphotoreceptor retinoid binding protein (Horai et al., 2015). In both groups of animal models of uveitis, there is a significant intraocular myeloid cell population which effects ocular tissue damage (Forrester et al., 1998).

T cells play a crucial role in the development of uveitis (Lee et al., 2014), but there is also increasing interest in the role of B lymphocytes (Smith et al., 2016; Wang et al., 2014). B cells could potentially contribute to the pathophysiology of uveitis in numerous ways that may be either regulatory or pro-inflammatory. B cells produce antibody and secretion of antibody (Adamus et al., 2006; Grewal et al., 2014), immune complex formation and subsequent activation of innate effector mechanisms, such as the complement system, has been implicated in both experimental and human disease (Marak et al., 1979; Vergani et al., 1986).

The local integration of immune responses can further condition the course of disease. Ultimately this can lead to the formation of ELS, focal aggregations of lymphocytes that develop in non-lymphoid tissues in response to chronic disease processes such as autoimmunity, inflammation, infection, neoplasia and transplant rejection (Drayton et al., 2006; Jones et al., 2016). ELS have similarities to secondary lymphoid organs including focal aggregations of B cells and T cells (in adjacent but anatomically distinct areas of tissue) and follicular dendritic cell networks, resembling the germinal centre-containing lymphoid follicles of SLOs. These structures have been reported to have many important properties in relation to both experimental and human disease, including being sites of auto-antibody production (Humby et al., 2009; Pitzalis et al., 2014). They have not been specifically reported in human uveitis, although diffuse and focal B cell and plasma cell infiltrates have been seen within the human aqueous and vitreous humor and the uveoretina on immunoprofiling (George et al., 1997; Kim et al., 1987; Lubin et al., 1980; Sabates et al., 1979).

This study has several limitations. Chorioretinal biopsy is only performed rarely and predominantly to diagnose neoplasia, so obtaining informative specimens was difficult. The samples examined in this study came from patients with severe and persistent disease, and experience indicates that the commonest reason for enucleation/evisceration would have been for intractable pain, although this was not always indicated in the pathology reports. We were therefore restricted to a small number of subjects, using tissue that was not obtained at different stages of disease and with very little clinical information. Tissue was likely not taken on the basis of inflammation, but on the basis of pain related to other complications. But because the development of ELS are associated with poor prognosis in multiple sclerosis (Choi et al., 2012), it remained important to investigate whether similar structures could develop in the immune privileged sites of the eye. We reviewed 200 reports including the word uveitis and studied 15 specimens in detail, finding ELS in 3. The data demonstrate that it is common for there to be a significant B cell presence within the immune cell infiltrates within the eye in severe uveitis. It is most often diffuse (Supplementary Fig. 1A), with or without additional focal features and, in this series, it is most evident within the uvea (Fig. 1 and Supplementary Fig. 1). The immune cell infiltrate had features of ELS in 3 out of 15 eyes examined, and 3 out of 11 of eyes with B cell infiltration of any kind. ELS in ocular tissue has all the features that are associated with it in other tissues (Fig. 1) including BCL6 and AID which are both expressed within germinal centres by B cells undergoing the germinal centre reaction, somatic hypermutation and class switch recombination. ELS is therefore a rare complication of severe uveitis, despite the eye being immune-privileged in the normal state.

The immune cell infiltrate we observed was assessed at a single time point in a single eye, yet displayed features of ELS at several different stages of development, from clusters of lymphocytes without FDC networks or clear T/B cell segregation, to focal aggregations of T and B cells segregated into anatomically distinct zones with an FDC network and the GC markers BCL6 and AID (Fig. 2). This finding is consistent with the ELS existing as semi-autonomous micro-environments, dynamic structures that develop and dissipate independently during the chronic disease process. The long-term presence of CD4 and CD8 cells (Fig. 4) that always accompanies B cell infiltrate likely relates to both disease persistence, and to the role that these cells play regulating immune responses in late disease, as shown in different animal models (Boldison et al., 2014; Silver et al., 2015).

It was also noted that in human uveitis the uvea is the ocular tissue predominantly affected by the immune cell infiltrate, with only sparse lymphocytes visible within the retina (Table 2). This contrasts with the data from a TCR transgenic animal model (Kielczewski et al., 2016), where ELS lesions develop within the retina as part of a process of spontaneous autoimmune uveitis. This discrepancy may relate to differences in the choroid in the mouse compared with human uveitis, or to chronicity of the underlying disease process in the human subjects compared with the relatively early timepoints at which EAU is usually studied. The anatomy of the choroid differs between mouse and man, in that the choroid is much thicker in humans and thus potentially more conducive to providing a suitable microenvironment for the development of ELS. A parallel could be drawn between uveitis and MS, where ELS have been demonstrated within the meninges rather than the neural brain parenchyma. Another possibility is that persistent human uveitis is maintained by reactivity to a different set of ocular autoantigens than is studied in animal experiments and that these have a different anatomical distribution.

Examining inflammatory genes that are selectively upregulated in ocular tissue, obtained from patients with persistent inflammation, confirms that these encompass a broad range of immunological function. One striking finding is the overrepresentation of MS4A1 which is the gene for CD20, the target of Rituximab. Therapy with this biologic has been reported in a number of small case studies, where it has been efficacious (Heiligenhaus et al., 2011; Lasave et al., 2018). Supporting our observation that the mRNA for CCL18 is increased, CCL18 has been reported in uveitis in human samples of aqueous humor in 12/30 samples (Abu El-Asrar et al., 2004). This chemokine has been associated with the accumulation of non-Foxp3 positive T regulatory cells (Chenivesse et al., 2012). CD14 is co-factor for LPS binding and is expressed as a GPI-linked protein by monocytes and macrophages. In uveitis, CD14^++^CD16^+^ monocytes are enriched in the circulation of patients with uveitis, when these patients are treated with glucocorticoids (Liu et al., 2015). The high level of CD14 detected may therefore arise as a result of extensive diffuse infiltration with monocytes (Fig. 3A). Fibronectin has been highlighted as an important molecule in recurrent equine uveitis where it has been associated with extracellular tissue remodelling. Levels of the protein were reduced in the vitreous, but in tissue sections it could be visualised in a dispersed pattern associated with a disintegration of the inner limiting membrane (Deeg et al., 2011). Our finding that fibronectin mRNA remains highly upregulated in the ocular tissue of patients with persistent uveitis suggests that late in the disease there continues to be significant disruption of normal extracellular matrix homeostasis, and this may accompany important changes associated with the stroma (Barone et al., 2016).

In summary, this study of eyes enucleated or eviscerated from patients with a diagnosis of uveitis demonstrates that when leukocytes are present in the tissue, T and B cell infiltration is common. ELS formation is a feature in a minority of cases and this provides a rationale for enhanced depth imaging optical coherence tomography in patients with uveitis as a methodology for a more systematic survey of the anatomical changes that accompany uveitis (Mrejen and Spaide, 2013), especially sub-retinal changes. Gene-expression in the affected tissue is consistent with ongoing active inflammation, and the dynamic nature of this process is further underlined by the different stages of ELS development that can be seen, even within the same eye. If this can be coupled with labelled cell imaging it may provide prognostic information to guide the use of immunotherapeutic regimens.

## Grant support

Supported by the Wellcome Trust (WT101777AIA).

## References

[bib1] Abu El-Asrar A.M., Struyf S., Descamps F.J., Al-Obeidan S.A., Proost P., Van Damme J., Opdenakker G., Geboes K. (2004). Chemokines and gelatinases in the aqueous humor of patients with active uveitis. Am. J. Ophthalmol..

[bib2] Adamus G., Webb S., Shiraga S., Duvoisin R.M. (2006). Anti-recoverin antibodies induce an increase in intracellular calcium, leading to apoptosis in retinal cells. J. Autoimmun..

[bib3] Aloisi F., Pujol-Borrell R. (2006). Lymphoid neogenesis in chronic inflammatory diseases. Nat. Rev. Immunol..

[bib4] Barone F., Gardner D.H., Nayar S., Steinthal N., Buckley C.D., Luther S.A. (2016). Stromal fibroblasts in tertiary lymphoid structures: a novel target in chronic inflammation. Front. Immunol..

[bib5] Boldison J., Chu C.J., Copland D.A., Lait P.J.P., Khera T.K., Dick A.D., Nicholson L.B. (2014). Tissue-resident exhausted effector memory CD8+ T cells accumulate in the retina during chronic experimental autoimmune uveoretinitis. J. Immunol..

[bib6] Boldison J., Khera T.K., Copland D.A., Stimpson M.L., Crawford G.L., Dick A.D., Nicholson L.B. (2015). A novel pathogenic RBP-3 peptide reveals epitope spreading in persistent Experimental Autoimmune Uveoretinitis. Immunology.

[bib7] Canete J.D., Celis R., Moll C., Izquierdo E., Marsal S., Sanmarti R., Palacin A., Lora D., de la Cruz J., Pablos J.L. (2009). Clinical significance of synovial lymphoid neogenesis and its reversal after anti-tumour necrosis factor alpha therapy in rheumatoid arthritis. Ann. Rheum. Dis..

[bib8] Caspi R.R. (2010). A look at autoimmunity and inflammation in the eye. J. Clin. Investig..

[bib9] Chenivesse C., Chang Y., Azzaoui I., Ait Yahia S., Morales O., Plé C., Foussat A., Tonnel A.-B., Delhem N., Yssel H., Vorng H., Wallaert B., Tsicopoulos A. (2012). Pulmonary CCL18 recruits human regulatory T cells.

[bib10] Choi S.R., Howell O.W., Carassiti D., Magliozzi R., Gveric D., Muraro P.A., Nicholas R., Roncaroli F., Reynolds R. (2012). Meningeal inflammation plays a role in the pathology of primary progressive multiple sclerosis. Brain.

[bib11] Corsiero E., Carlotti E., Prediletto E., Jagemann L., Pitzalis C., Bombardieri M. (2016). The role of somatic hypermutation and N-glycosylation in the anti-NET immunoreactivity of RA synovial monoclonal antibodies. Eur. J. Immunol..

[bib12] Deeg C.A., Eberhardt C., Hofmaier F., Amann B., Hauck S.M. (2011). Osteopontin and fibronectin levels are decreased in vitreous of autoimmune uveitis and retinal expression of both proteins indicates ECM Re-modeling. PLoS One.

[bib13] Deeg C.A., Ehrenhofer M., Thurau S.R., Reese S., Wildner G., Kaspers B. (2002). Immunopathology of recurrent uveitis in spontaneously diseased horses. Exp. Eye Res..

[bib14] Deschenes J., Murray P.I., Rao N.A., Nussenblatt R.B. (2008). International uveitis study group (IUSG) clinical classification of uveitis. Ocul. Immunol. Inflamm..

[bib15] Drayton D.L., Liao S., Mounzer R.H., Ruddle N.H. (2006). Lymphoid organ development: from ontogeny to neogenesis. Nat. Immunol..

[bib16] Epps S.J., Boldison J., Stimpson M.L., Khera T.K., Lait P.J.P., Copland D.A., Dick A.D., Nicholson L.B. (2018). Re-programming immunosurveillance in persistent non-infectious ocular inflammation. Prog. Retin. Eye Res..

[bib17] Forrester J.V., Huitinga I., Lumsden L., Dijkstra C.D. (1998). Marrow-derived activated macrophages are required during the effector phase of experimental autoimmune uveoretinitis in rats. Curr. Eye Res..

[bib18] Forrester J.V., Kuffova L., Dick A.D. (2018). Autoimmunity, autoinflammation, and infection in uveitis. Am. J. Ophthalmol..

[bib19] George R.K., Chan C.C., Whitcup S.M., Nussenblatt R.B. (1997). Ocular immunopathology of Behcet's disease. Surv. Ophthalmol..

[bib20] Germain C., Gnjatic S., Dieu-Nosjean M.C. (2015). Tertiary lymphoid structure-associated B cells are key players in anti-tumor immunity. Front. Immunol..

[bib21] Grewal D.S., Fishman G.A., Jampol L.M. (2014). Autoimmune retinopathy and antiretinal antibodies: a review. Retina- J. Retin. Vitr. Dis..

[bib22] Heiligenhaus A., Miserocchi E., Heinz C., Gerloni V., Kotaniemi K. (2011). Treatment of severe uveitis associated with juvenile idiopathic arthritis with anti-CD20 monoclonal antibody (rituximab). Rheumatology.

[bib23] Horai R., Zárate-Bladés, Carlos R., Dillenburg-Pilla P., Chen J., Kielczewski Jennifer L., Silver Phyllis B., Jittayasothorn Y., Chan C.-C., Yamane H., Honda K., Caspi Rachel R. (2015). Microbiota-dependent activation of an autoreactive T cell receptor provokes autoimmunity in an immunologically privileged site. Immunity.

[bib24] Howell O.W., Reeves C.A., Nicholas R., Carassiti D., Radotra B., Gentleman S.M., Serafini B., Aloisi F., Roncaroli F., Magliozzi R., Reynolds R. (2011). Meningeal inflammation is widespread and linked to cortical pathology in multiple sclerosis. Brain.

[bib25] Humby F., Bombardieri M., Manzo A., Kelly S., Blades M.C., Kirkham B., Spencer J., Pitzalis C. (2009). Ectopic lymphoid structures support ongoing production of class-switched autoantibodies in rheumatoid synovium. PLoS Med..

[bib26] Jones G.W., Hill D.G., Jones S.A. (2016). Understanding immune cells in tertiary lymphoid organ development: it is all starting to come together. Front. Immunol..

[bib27] Kielczewski J.L., Horai R., Jittayasothorn Y., Chan C.-C., Caspi R.R. (2016). Tertiary lymphoid tissue forms in retinas of mice with spontaneous autoimmune uveitis and has consequences on visual function. J. Immunol..

[bib28] Kim M.K., Chan C.C., Belfort R., Farah M., Burnier M.P., Nussenblatt R.B., Kuwabara T., Palestine A.G. (1987). Histopathologic and immunohistopathologic features of subretinal fibrosis and uveitis syndrome. Am. J. Ophthalmol..

[bib29] Kleinwort K.J.H., Amann B., Hauck S.M., Feederle R., Sekundo W., Deeg C.A. (2016). Immunological characterization of intraocular lymphoid follicles in a spontaneous recurrent uveitis model. Investig. Ophthalmol. Vis. Sci..

[bib30] Lasave A.F., You C.Y., Ma L.N., Abusamra K., Lamba N., Navarro M.V., Meese H., Foster C.S. (2018). Long-term outcomes of rituximab therapy in patients with noninfectious posterior uveitis refractory to conventional immunosuppressive therapy. Retina.

[bib31] Law C.W., Chen Y.S., Shi W., Smyth G.K. (2014). voom: precision weights unlock linear model analysis tools for RNA-seq read counts. Genome Biol..

[bib32] Lee R.W.J., Nicholson L.B., Sen H.N., Chan C.C., Wei L., Nussenblatt R.B., Dick A.D. (2014). Autoimmune and autoinflammatory mechanisms in uveitis. Semin. Immunopathol..

[bib33] Liu B., Dhanda A., Hirani S., Williams E.L., Sen H.N., Estrada F.M., Ling D., Thompson I., Casady M., Li Z., Si H., Tucker W., Wei L., Jawad S., Sura A., Dailey J., Hannes S., Chen P., Chien J.L., Gordon S., Lee R.W.J., Nussenblatt R.B. (2015). CD14(++)CD16(+) monocytes are enriched by glucocorticoid treatment and are functionally attenuated in driving effector T cell responses. J. Immunol..

[bib34] Lubin J.R., Albert D.M., Weinstein M. (1980). 65 Years of sympathetic ophthalmia - clinicopathologic review of 105 cases (1913-1978). Ophthalmology.

[bib35] Magliozzi R., Howell O., Vora A., Serafini B., Nicholas R., Puopolo M., Reynolds R., Aloisi F. (2007). Meningeal B-cell follicles in secondary progressive multiple sclerosis associate with early onset of disease and severe cortical pathology. Brain.

[bib36] Marak G.E., Wacker W.B., Rao N.A., Jack R., Ward P.A. (1979). Effects of complement depletion on experimental allergic uveitis. Ophthalmic Res..

[bib37] Mrejen S., Spaide R.F. (2013). Optical coherence tomography: imaging of the choroid and beyond. Surv. Ophthalmol..

[bib38] Pikor N.B., Cupovic J., Onder L., Gommerman J.L., Ludewig B. (2017). Stromal cell niches in the inflamed central nervous system. J. Immunol..

[bib39] Pitzalis C., Jones G.W., Bombardieri M., Jones S.A. (2014). Ectopic lymphoid-like structures in infection, cancer and autoimmunity. Nat. Rev. Immunol..

[bib40] Rao D.A., Gurish M.F., Marshall J.L., Slowikowski K., Fonseka C.Y., Liu Y.Y., Donlin L.T., Henderson L.A., Wei K., Mizoguchi F., Teslovich N.C., Weinblatt M.E., Massarotti E.M., Coblyn J.S., Helfgott S.M., Lee Y.C., Todd D.J., Ykerk V.P.B., Goodman S.M., Pernis A.B., Ivashkiv L.B., Karlson E.W., Nigrovic P.A., Filer A., Buckley C.D., Lederer J.A., Raychaudhuri S., Renner M.B.B. (2017). Pathologically expanded peripheral T helper cell subset drives B cells in rheumatoid arthritis. Nature.

[bib41] Ritchie M.E., Phipson B., Wu D., Hu Y., Law C.W., Shi W., Smyth G.K. (2015). Limma powers differential expression analyses for RNA-sequencing and microarray studies. Nucleic Acids Res..

[bib42] Sabates R., Smith T., Apple D. (1979). Ocular histo-pathology in juvenile rheumatoid-arthritis. Ann. Ophthalmol..

[bib43] Shechter R., London A., Schwartz M. (2013). Orchestrated leukocyte recruitment to immune-privileged sites: absolute barriers versus educational gates. Nat. Rev. Immunol..

[bib44] Silver P.B., Horai R., Chen J., Jittayasothorn Y., Chan C.-C., Villasmil R., Kesen M.R., Caspi R.R. (2015). Retina-specific T regulatory cells bring about resolution and maintain remission of autoimmune uveitis. J. Immunol..

[bib45] Smith J.R., Stempel A.J., Bharadwaj A., Appukuttan B. (2016). Involvement of B cells in non-infectious uveitis. Clin. Transl. Immunol..

[bib46] Trusko B., Thorne J., Jabs D., Belfort R., Dick A., Gangaputra S., Nussenblatt R., Okada A., Rosenbaum J., Standardization Uveitis N. (2013). The standardization of uveitis nomenclature (SUN) project development of a clinical evidence base utilizing informatics tools and techniques. Methods Inf. Med..

[bib47] Vergani S., Dimauro E., Davies E.T., Spinelli D., Mielivergani G., Vergani D. (1986). Complement activation in uveitis. Br. J. Ophthalmol..

[bib48] Wang R.-X., Yu C.-R., Dambuza I.M., Mahdi R.M., Dolinska M., Sergeey Y.V., Wingfield P.T., Kim S.-H., Egwuagu C.E. (2014). Interleukin-35 induces regulatory B cells that suppress autoimmune disease. Nat. Med..

[bib49] Warnes G.R., Ben Bolker, Lodewijk Bonebakker, Gentleman R., Huber Andy Liaw W., Thomas Lumley, Martin Maechler, Arni Magnusson, Moeller S., Marc Schwartz, Bill (2019). gplots: Various R Programming Tools for Plotting Data. R package version 3.0.1.1. Venables.

