# Supplementary material for: Features of ectopic lymphoid-like structures in human uveitis
Source: Exp Eye Res. 2020 Feb;191:107901. doi: 10.1016/j.exer.2019.107901 (PMC7029346; doi:10.1016/j.exer.2019.107901)
Supplement: Multimedia component 1 [file mmc1.docx]

Supplementary Table 1: Primary antibodies for immunohistochemistry

| Antibody specificity and source | Pre-treatment pH | Dilution | Linker | Type | Clone |
| --- | --- | --- | --- | --- | --- |
| CD3, Dako | High | Ready to use | None | Rabbit polyclonal | N/A |
| CD4, Leica | High | 1:20 | None | Mouse monoclonal | 4B12 |
| CD8, Dako | High | 1:200 | None | Mouse monoclonal | C8/144B |
| CD20, Dako | High | Ready to use | Mouse linker | Mouse monoclonal | L26 |
| CD21, Dako | Low | 1:20 | Mouse linker | Mouse monoclonal | 1F8 |
| CD23, Dako | Low | Ready to use | Mouse linker | Mouse monoclonal | DAK-CD23 |
| CD68, Dako | High | 1:200 | Mouse linker | Mouse monoclonal | PG-M1 |
| CD138, Dako | High | 1:50 | Mouse linker | Mouse monoclonal | MI15 |
| BCL6, Dako | High | 1:40 | Mouse linker | Mouse monoclonal | PG-B6p |
| AID, Life Sciences | High | 1:500 | Mouse linker | Mouse monoclonal | ZA001 |
